# Systemic Capillary Leak Syndrome as a Paraneoplastic Syndrome

**DOI:** 10.7759/cureus.60923

**Published:** 2024-05-23

**Authors:** Bernardo Silva, Vasco Gaspar, Cláudia Alves, Maria Isabel Andrade, Jesennia Chinchilla Mata

**Affiliations:** 1 Internal Medicine, Hospital Distrital de Santarém, Santarém, PRT; 2 Anatomical Pathology, Hospital Distrital de Santarém, Santarém, PRT

**Keywords:** paraneoplastic syndrome, non-hodgkin lymphoma, anasarca, systemic capillary leak syndrome, diffuse large b-cell lymphoma

## Abstract

Systemic capillary leak syndrome (SCLS) is a rare entity that is frequently idiopathic or, rarely, associated with infections, autoimmune diseases, drugs, surgery, and cancer. Several cancers can directly cause SCLS, although it is very uncommon as the inaugural presentation of a non-Hodgkin lymphoma. We report a case of SCLS as a paraneoplastic syndrome which revealed a large B-cell lymphoma, a non-Hodgkin lymphoma of B-cell origin.

## Introduction

Systemic capillary leak syndrome (SCLS) is an infrequent condition described for the first time by Clarkson et al. in 1960. It is frequently idiopathic, characterized by a classic triad of hypoalbuminemia, haemoconcentration, and hypotension, or, rarely, associated with infections, autoimmune diseases, drugs, surgery, and cancer [1,2]. Several cancers can directly cause SCLS. However, it is very uncommon as the inaugural presentation of a lymphoma and even more rare as the way of disclosing a non-Hodgkin lymphoma, typically diagnosed through immunophenotypic studies of biopsies taken from suspicious lymph nodes [1,2].

Here, we report a case of SCLS as a paraneoplastic syndrome which revealed a diffuse large B-cell lymphoma, allowing for the initiation of directed therapy.

## Case presentation

**Figure 1:**
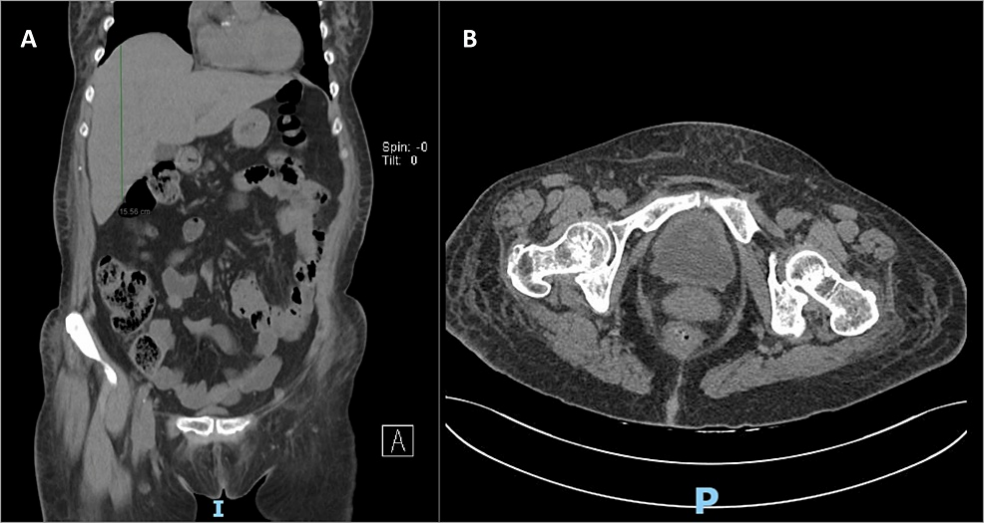
Abdominal CT scan. A: Hepatomegaly. B: Bladder diffuse thickness.

A 77-year-old woman with a medical history of hypertension, type 2 diabetes mellitus, and non-obstructive hypertrophic cardiomyopathy was admitted to the emergency department with asthenia, anorexia, and tiredness with two weeks of evolution. Upon physical inspection, the patient had no relevant findings. Blood samples revealed anemia (hemoglobin: 10.5 g/dL), reduced hematocrit (32.2%), leucocytosis (11.7 × 10^9^/L), neutrophilia (83%), increased C-reactive protein (CRP) (15 mg/dL), acute renal failure (urea: 70 mg/dL and creatinine 1.6: mg/dL), and cytocholestasis (aspartate transaminase: 50 U/L, alkaline phosphatase: 309 U/L, and glutamyl transferase: 189 U/L). Urinalysis revealed the presence of leukocytes. The computed tomography (CT) scan showed hepatomegaly (Figure 1A), bladder with diffuse thickness (Figure 1B), and absence of adenopathies.

Therefore, in the presence of leucocytosis, neutrophilia, increased CRP, leukocyturia, and increased creatinine levels, the diagnoses of pyelonephritis and acute renal failure were assumed, and the patient was admitted to the home hospital department and started treatment with ceftriaxone.

As there was no clinical improvement after five days, the medical assistant switched antibiotic treatment from ceftriaxone to ertapenem. However, after three days of this antibiotic, the patient developed fever, drowsiness, oliguria, and anasarca and was transferred to the internal medicine ward. On physical examination, the patient revealed hypotension (87/38 mmHg), dyspnea, and anasarca signs. Relevant laboratory data were pancytopenia, worsening acute renal failure (urea: 92 mg/dL, creatinine: 2.5 mg/dL), increased serum lambda and kappa chains, increased lactate dehydrogenase (493 U/L), and increased inflammatory parameters (CRP: 26.4 mg/dL) though with no leucocytosis. There was no evidence of hemoconcentration. Serum protein and albumin (albumin: 2.1 g/dL) were significantly reduced, without proteinuria. Urine immunofixation was negative for free light chains. The point-of-care ultrasound revealed regular cardiac function and the inferior vena cava non-dilated and collapsible with inspirations despite the anasarca. As hemocultures and urocultures were negative, a generalized inflammatory process manifesting as SCLS was assumed.

**Figure 2:**
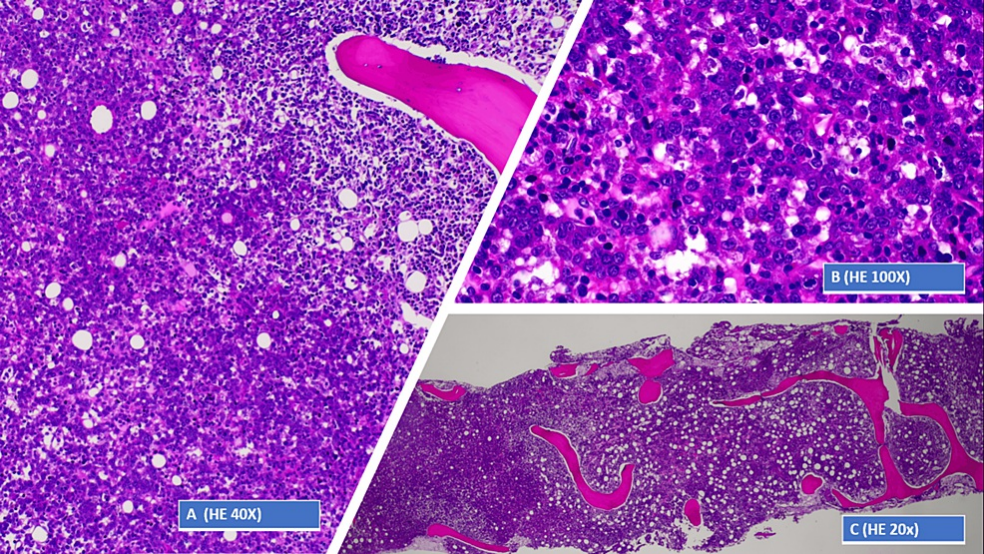
Bone marrow biopsy. A: Bone marrow biopsy - 40× view of hematoxylin and eosin-stained section. B: Bone marrow biopsy - 100× view of hematoxylin and eosin-stained section. C: Bone marrow biopsy - 20× view of hematoxylin and eosin-stained section.

In this context, the patient was started on methylprednisolone and, thinking of a possible paraneoplastic syndrome, in the presence of hematological findings (pancytopenia, increased serum lambda and kappa chains, and hepatomegaly) and without evidence of a solid tumor on the CT scan, a bone marrow biopsy was performed. This examination revealed hypercellularity due to interstitial, nodular, and diffuse infiltration, consisting of large cells (Figure 2A) with cleaved nuclei, vesicular chromatin, and sometimes evident nucleoli (Figure 2B), constituting 50% of the total nucleated cellularity (Figure 2C).

**Figure 3:**
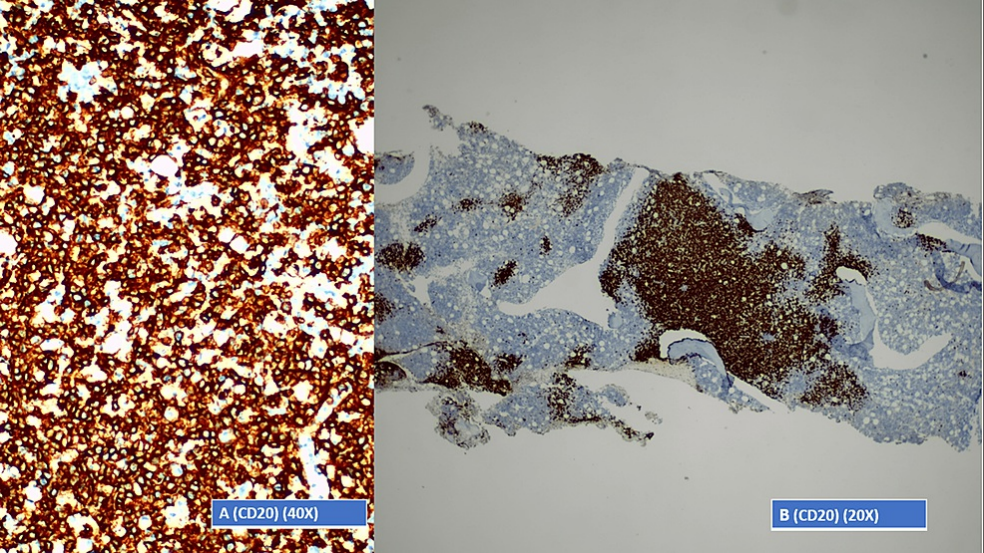
Bone marrow biopsy. A: Bone marrow - 40× view of the immunohistochemical study. B: Bone marrow - 20× view of the immunohistochemical study.

In the immunohistochemical (IHC) study, the neoplastic cells were positive for CD20 (Figures 3A, 3B), BCL-2, MUM1, and CD5 and negative for CD10, BCL-6, Cyclin D1, CD3, CD30, and ALK1. Morphological and IHC findings favored the diagnosis of medullary infiltration by a non-Hodgkin lymphoma of large B cells (CD20+), with aberrant CD5 staining, which correlated with clinical and laboratory findings, making the diagnosis of a diffuse large B-cell lymphoma (DLBCL), a non-Hodgkin lymphoma of B-cell origin.

Although the patient had a significant improvement with corticosteroid therapy initiation, after starting R-CHOP (rituximab, cyclophosphamide, doxorubicin, vincristine, and corticosteroid) treatment, she developed sepsis and died a few days later.

## Discussion

SCLS is a rare entity whose exact incidence is unclear, mainly because it is very challenging to diagnose [3]. It can be idiopathic or, rarely, associated with autoimmune diseases, sepsis, viral hemorrhagic fever, surgery, ovarian hyperstimulation syndrome, hemophagocytic lymphohistiocytosis, drugs, and cancer [1,2].

SCLS may be suspected when a characteristic triad occurs, i.e., hypotension, hypoalbuminemia, and paradoxical hemoconcentration [1]. This results from increased capillary permeability, leading to endothelial damage and fluid loss from the intravascular compartment into interstitial space. Nevertheless, it is essential to realize that it mimics several other diseases that are very challenging to diagnose and treat, being frequently a diagnosis of exclusion [1,2].

The clinical presentation of SCLS in cancer patients is identical to that observed in idiopathic forms and includes dyspnea, ascites, and edema as a consequence of capillary leaks [3].

Several cancers can directly cause SCLS. However, in a systematic review, it is most often associated with hematologic malignancy, and most patients have non-Hodgkin lymphomas [2,3]. Concerning the prognosis of this syndrome in cancer patients, studies suggest that hematologic malignancies are associated with an increased risk for mortality [3]. Therefore, fast diagnosis is essential to control the development of symptoms, avoid clinical deterioration, and initiate the appropriate cancer treatment.

DLBCL accounts for 30-58% of non-Hodgkin lymphomas with a five-year survival duration ranging between 30% and 80% [4,5]. It comprises intermediate and high-grade B-cell lymphomas, which, in up to one-third of cases, arise from extranodal sites or spread to extranodal organs, particularly the liver, peritoneum, bone marrow, central nervous system, and pleura, sometimes obscuring the primary site of origin and making it harder to diagnose [6]. In fact, the diagnosis is ideally made from an excisional biopsy of an abnormally enlarged, suspicious-appearing lymph node, which sometimes cannot be found [7].

Although patients may present with various clinical symptoms, they typically have progressive lymphadenopathy, extranodal disease, and specific B symptoms (weight loss, night sweats, and fever). Rashes on the skin, fatigue, pruritus, fever, anasarca, and effusions are less commonly presenting features [8].

The disease is aggressive, and patients typically necessitate immediate treatment. Otherwise, it can result in death within a few weeks. Although most patients present in an advanced stage of the disease, more than 60% can be cured with R-CHOP immunochemotherapy, which shows the importance of the diagnosis [9].

Despite the outcome of the case reported, we bring to attention the unusual and rare nature of SCLS presentation and how it was essential to make the diagnosis of DLBCL as it led to a search for a primary cause for the symptoms.

## Conclusions

The lack of understanding of the underlying mechanisms causing SCLS and proper treatment guidelines, especially in cancer patients, makes diagnosing and treating this condition challenging. However, it is vital to bear this case in mind so that in the future, in a similar situation, an early diagnosis can be made and patients can start directed treatment for cancer and, subsequently, SCLS sooner.
